# Land Use Change Assessment and Water Quality of Ephemeral Ponds for Irrigation in the North West Province, South Africa

**DOI:** 10.3390/ijerph15061175

**Published:** 2018-06-05

**Authors:** Frederick Asare, Lobina G. Palamuleni, Tabukeli Ruhiiga

**Affiliations:** School of Geo and Spatial Sciences, Department of Geography and Environmental Sciences, North West University, Private Bag X2046, Mmabatho 2735, South Africa; fredderickasare@gmail.com (F.A.); tabukeli.ruhiiga@nwu.ac.za (T.R.)

**Keywords:** crop production, land cover change, irrigation, water quality

## Abstract

In the semi-arid environments of the North West province of South Africa the amount, timing, and distribution of rainfall is irregular, while water accessibility is a key factor in production. In line with this, a study was conducted to assess the impact of land use change on water quality and water depth within the sub-catchment areas of ephemeral ponds. To determine land use dynamics, 2004 and 2013 Landsat images were classified using maximum likelihood algorithm. Pond water quality was analysed for physical, chemical, and microbiological parameters using standard the American Public Health Association (APHA) methods. Multiple linear regression models were computed to determine relationships between land use changes and water quality parameters. Results revealed a reduction in grass cover, whereas built-up areas increased at the expense of bare land. All the values for the physical characteristics were higher than the recommended Department of Water Affairs (DWAF) and Food and Agriculture Organisation (FAO) limits, but chemical parameters, except cadmium, were within limits. Regression showed that bare areas have a positive effect on *Escherichia coli* (*E. coli*) in ephemeral pond water. The study highlights the suitability of pond water for irrigation to increase crop production and the effects of land use changes on ecosystems as critical for proper catchment planning, water resource management, and food security.

## 1. Introduction

Land use within catchment areas has great impacts on the water quality of water bodies. This arises as a result of the process interactions between land and water, which in turn create symbiotic relations across time. Understanding these process interactions provides the theoretical base for investigating land use/land cover change under the parallel impact of natural forces and human activities. Ephemeral ponds exist on a seasonal basis within particular catchments. Assessing the water quality of such ponds should provide the means to analyse potential for small-scale irrigation agriculture. Naturally therefore, land use changes that take place within such catchments also impact on water quality within such ponds. This occurs as a result of material exchanges between the ponds and land uses within close proximity.

Water quality may degrade due to changes in the land use patterns within the catchment as human activities increase [1,2,3]. Changes in land use and land management practices are critical in influencing factors behind the alteration of the hydrological systems. These in turn may lead to changes in runoff [4] as well as the water quality [5]. Zamani [6] and Chow et al. [7] reported that activities such as intensive agriculture, industry, and high concentrations of human settlement tend to pollute water bodies. These pollutants may also influence the quality of ephemeral pond water by virtue of their location in a catchment area. Divya and Belagali [8] added that chemical fertilizers affect the quality of water, and hence effective management of fertilizer application in a catchment area has to be done. Excessive use of fertilizers and chemicals pollute the soil. Land use/land cover (LULC) changes, especially those driven by human activities, are some of the most important components of global environmental change [9]. Land use change in catchments results from a variety of natural and anthropogenic sources. The conversion of vegetated areas into built-up areas or grassland disrupts the hydrological cycle of a drainage basin by altering the balance between rainfall and evaporation and, consequently, the runoff response of the area [10]. Bare land accelerates nutrient and chemical runoff into ponds. These in turn negatively impact on water quality. In addition, where there are grazing animals and ponds are used for watering, a lot of excreta can enter the ponds through runoff or directly, resulting into pond water contamination. Moreover, built-up areas have profound effects on the quality of water in the ponds. Built-up areas are associated with developments such as industries, human settlements and mining etc. As a result, effluents from these areas mix with the runoff and storm water and eventually ending up in water bodies, including the ephemeral ponds. Built-up areas form concrete surfaces and speed up the flow of water to ponds.

It should, however, be noted that not all land use and land cover changes place negative effects on water quality. Forest establishment, planting of fodder, and plantation agriculture improve the quality of wetlands because these practices require lower amounts of fertilizers and pesticides, and hence there is less pollution of the water resources [11]. Moreover, vegetation cover in general traps silt, manure, fertilizers, and rubbish, and prevents them from flowing into the ephemeral pond water. This helps to reduce pollution of the pond water. While land use/land cover changes are a long-term phenomenon, ephemeral ponds on the other hand are short-term periodic features. There is, however, an inherent relationship between the two because the latter appears practically every year. Secondly, ephemeral ponds are a result of natural hydrological forces, which also have a direct impact onto the LULC changes. In reality therefore, the difference between long-term and short-term factors of land cover changes in the understanding of natural and human impacts becomes diminished.

Most rural communities in South Africa experience serious water shortages due to low rainfall and high evapotranspiration rates. The study area, the Vryburg District in the North West province of South Africa, is a semi-arid area. Ephemeral ponds are small, isolated deep depressions that only fill up during the short rainfall events. They are water bodies that occupy depressions in the local terrain and are associated with impeded drainage. These ponds form part of the wetlands in general, including other water bodies such as the vernal ponds, bogs, mangroves, temporary pools, and seasonal wetlands [12]. The ponds tend to be short-lived but there is no scientific agreement as to when alternative terms should apply. However, for the purposes of this study, a cut-off date of six months is hereby proposed. These ephemeral ponds are characterised by variability in location, surface area, longevity, water quality, volume of water stored, and mean depth. The longevity of these ponds varies between a few days to several months, depending on some of the parameters mentioned earlier. Much of the water is lost through high temperatures that cause high evaporation rates. In addition, natural seepage into groundwater adds to water loss. These ponds are essentially short-lived water reservoirs located in semi-arid areas, where the potential for irrigation farming has so far not been assessed. Due to the short retention capacity of the ponds, they could be possibly used for small-scale agriculture such as such the growing of vegetables and field crops (maize, peanut and soybeans). The hypothesis for this study was that the use of ephemeral pond water for irrigation would significantly improve and raise the level of production, and consequently the livelihood outcomes, which cannot be in isolation of the other livelihood assets. The aim of the study was, therefore, to explore the dynamics of ephemeral ponds in relation to land-use in the surrounding vicinity in order to analyse their potential for improving small-scale irrigation. The primary objectives were (1) to assess the water quality of ephemeral ponds in Vryburg District; (2) to investigate the land use change in the study area; and (3) to identify the relationship(s) between land use land cover change and water quality. In line with this, it was hypothesized that the use of ephemeral pond water for irrigation could significantly improve the level of production, and consequently, the livelihood outcomes of the adjacent local communities.

## 2. Materials and Methods

### 2.1. Study Area

**Figure 1:**
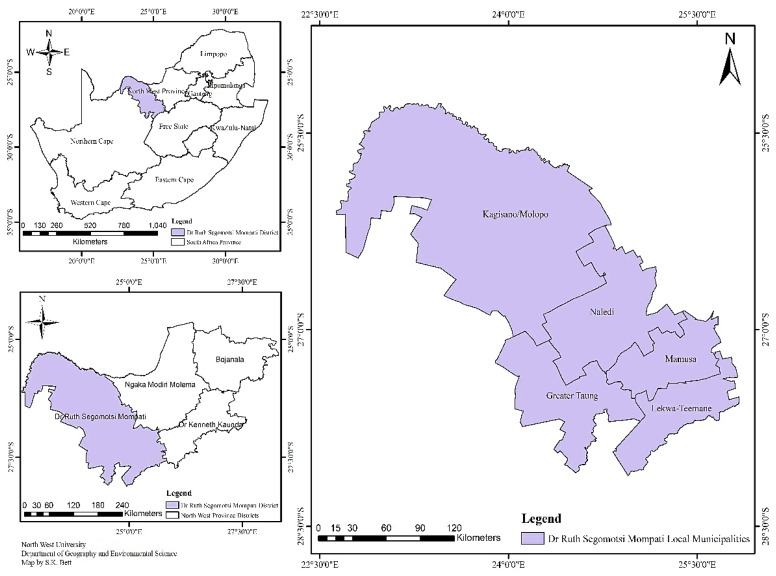
Map of the local municipalities of the study area.

The study area was Vryburg District, a municipal district found in the North West Province of South Africa. The area is located between 25°16′ S–28°6′ S and 22°38′ E–26°14′ E (Figure 1). The district is semi-arid and records a mean annual rainfall of around 410 mm. The area experiences summer rainfalls mainly from October to March, sometimes extending to April in the following year. The mean day temperature in summer is 33 °C and night temperature 18 °C. Winter is cool, with the mean night temperature sometimes being below 0 °C and that of the day reaching 19 °C. The relative humidity is between 64 and 66% in February and about 28–32% in July. Frost occurrence is a regular feature, appearing on between 31 and 60 days in winter. Also, the rate of evaporation exceeds that of precipitation in the district. The dominant biome is grassland savannah consisting of Kalahari Thornveld and Bushveld shrub vegetation [13]. The vegetation is of poor quality. Therefore, the majority of the area is used for grazing cattle, sheep, and goats, except for the Taung Irrigation Scheme that produces field crops. Agriculture is regarded as the major land use with mixed crop farming including barley, wheat, sunflower, maize, cotton, and nuts.

### 2.2. Data Sources

#### 2.2.1. Land Use Data

Landsat images, level 1G, were obtained from the South Africa National Space Agency (SANSA). A strategy for selecting Landsat imagery for the development of land use database for the study area was governed by the available multi-temporal images, vegetation phenology and image quality (cloudiness, haze). The data set was derived from Landsat satellite images spanning over a 10-year period (Landsat 5 Thematic Mapper of 24 April 2004 and Landsat 7 Enhanced Thematic Mapper+ (Landsat 7 ETM^+^) of 17 April 2013). The use of the shapefile of the study area and processed multi-temporal Landsat 7 ETM^+^ imagery assisted in the identification of the location and distribution, and area covered by each pond respectively and in order to determine their capacity. A total of 22 ponds were identified, but 5 were selected for this study, based on proximity to road, size and the longevity of the pond water. Apparently, there are restrictions in accessing ephemeral ponds located on private property in much of South Africa. A stratified random sampling to select the five ponds was therefore, the result of filtering. The Advanced Spaceborne Thermal Emission and Reflection Radiometer (ASTER) 30-m resolution digital elevation model (DEM) data was used to extract the slope length and height of each of the selected ponds as these determine the quantity of water that is collected in each pond. The DEM was also used to demarcate the sub-catchment area of each selected pond. Geographical information system (GIS) tools were used to calculate the area of each land use type within each pond sub-catchment. Based on that, the proportion of each land use area within each pond sub-catchment was then obtained.

#### 2.2.2. Characteristics of the Selected Ponds

The location of a pond, slope height, length, area covered by the pond, and its depth determine the amount of water it can store. These parameters affect the capacity of a pond and its subsequent suitability for irrigation. When a pond is located down a steep slope, a lot of water is collected by the slope and runs down quickly to be stored in the pond. This then contributes to the final volume of water in the pond. The water also spreads over a larger area. In addition, when the slope around a pond extends over a large area, more water moves down and collects in the pond.

In this work, a total of five ponds (herein designated A, B, C, D, and E) were studied. Ponds A, B, and C are situated on a long elevation between the Madibogo and Delareyville areas. Their formation was due to the high rainfall in the area, low water permeability, and the nature of the underlying rock. Pond A is situated at latitude 26°32′43″ S and longitude 25°03′01″ E. The water occupied an area of 37.8 ha. The maximum water depth in 2014 was 1.620 m. The highest point on the slope is 1365 m with an average slope height of 13.61 m above the water surface. It is bordered by a long slope that also contributes to the amount of water that collects in it. Pond B is located at latitude 26°53′52″ S and longitude 25°20′06″ E. The highest point on the slope is 1387 m. The average slope height is 18.3 m. The pond covered an area of 50.6 ha with a maximum depth of 2.5 m in 2014. Pond C is located at latitude 26°41′6″ S and longitude 25°24′34″ E. It is bordered by a steep slope at the north western part of the pond. The highest point of the slope is 1351 m with an average slope height of 7.7 m. The steep nature of the slope assists in the flow of runoff into the pond. In 2014, when measurements were taken, the water covered an area of 17.6 ha and a maximum depth of 2.3 m was recorded. Pond D is found between Pudimoe and Dryharts along the Taung–Vryburg road. It is located at latitude 27°16′52″ S and longitude 24°46′49″ E. The highest point on the DEM is 1264 m and the lowest is 1077 m. The average slope height is 39.5 m. In 2014, the height of the water column was 0.45 m with an area of 25.3 ha. Pond E can be found along the Vryburg–Setlagole road next to the Stella Junction. It is located at latitude 26°32′52″ S and longitude 24°52′28″ E. It is bordered by a long slope of the highest point of 1357 m. In 2014, the water covered an area of 61.2 ha and a depth of 2.15 m (Table 1).

**Figure 2:**
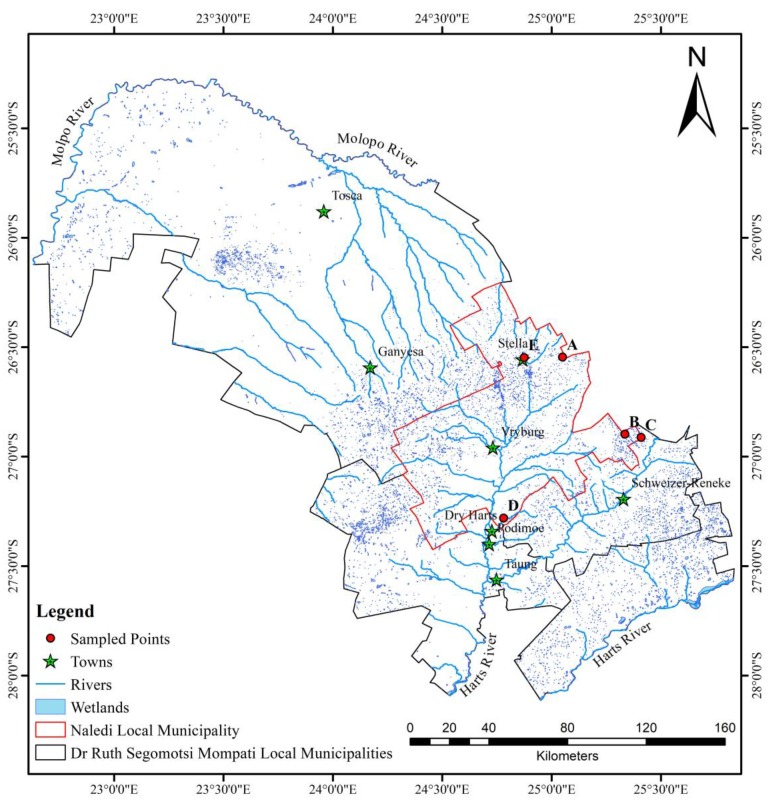
Distribution of wetlands and ephemeral ponds in the study area.

It should be noted that the Kagisano area is virtually devoid of ponds. This is mainly due to the dryness of the place. The area experiences relatively low rainfall. Furthermore, the underlying rock consists of dolomite and siltstone. Dolomite is responsible for the formation of groundwater hence rainwater infiltrates and accumulates underground. More ponds are formed around Stella, Schweitzer Reneke, and its surroundings. However, larger ponds are observed around Delareyville. This is due to the high rainfall around those areas and also the surrounding areas have rocks that consist of siltstone, andesite, and tillite that store water on the surface. Small ponds form in and around Taung, but due to the nature of the underlying rock, the water percolates to form groundwater. When rainfall intensity is above average for a particular season, the ponds start filling until they reach their maximum height in February. The longevity of the bigger ponds is prolonged, and thus they could be used for irrigation. The smaller ponds lose their water immediately after the rainy season (Figure 2).

#### 2.2.3. Water Quality

The distribution and the number of ponds depend on rainfall intensity and soil characteristics. During the periods of high rainfall, more ponds are formed and vary in size from a few hectares to about 60 hectares (Figure 2). The longevity of the ponds also ranges from a few days to about six months.

Water samples were collected from the five ephemeral ponds for analysis of physical, chemical, and microbiological parameters between March and April of 2014. The samples were collected in triplicate from the middle of the ponds just below the surface using 500-mL plastic bottles sterilized and neutralized with sodium thiosulfate. The samples were replicated to ensure accuracy of the data. Each bottle was filled to 90% full and then covered tightly. The bottles were kept in a cooler box at a temperature of between 4 and 10 °C for laboratory analysis. Table 2 shows the water quality parameters, measurements, and units.

The microbiological analysis was undertaken within 24 h. In situ measurements for temperature, pH (hydrogen potential), and electric conductivity (EC) were carried out using standard procedures [14] by way of field meters. Samples for chemical analysis were collected and taken to the laboratory. The chemical analysis included cations such as sodium (Na^+^), potassium (K^+^), magnesium (Mg^++^), calcium (Ca^++^), and cadmium (Cd^++^). The anions analysed included phosphate (PO_4_^−−−^), nitrate (NO_3_^−−^), and chloride (Cl^−^).

### 2.3. Data Analysis

#### 2.3.1. Land Use Mapping

Land use mapping and subsequent quantitative change detection requires geometric registration between image scenes, and radiometric rectification to adjust for differences in atmospheric conditions, viewing geometry and sensor noise and response [15,16]. To remove any geometric distortions in the Landsat 5 Thematic Mapper imagery, the first ETM+ image (2004) was registered to a Universal Transverse Mercator map projection (zone 35S, datum WGS84), using a nearest neighbour resampling technique. This was followed by image-to-image registration of the 2013 images to the 2004 image, utilising similar sets of ground control points with a root mean square error of less than one pixel. A subset of each of the five ponds to delineate the sub-catchment area for classification was followed by image enhancement using the histogram equalisation technique and identification of training sites. Classification of remote sensing data was done through the use of a maximum likelihood classification method. The advantage of the maximum likelihood algorithm is that it takes the variability of the classes into account by using the covariance matrix [16]. A 5 × 5 pixel majority filter was used to clean the classified images to the generalisation of the ephemeral ponds’ catchment maps. Change detection was done for 2004–2013 to get ‘from–to’ information of changes in land use and land cover in the study area, using the post-classification cross-tabulation approach [15].

#### 2.3.2. Accuracy Assessment

In this study, field exercise facilitated the identification of ground reference samples for use in the assessment of the accuracy of classified images. Furthermore, to correlate spectral features of the image with features on the ground, the field exercise was conducted during the dry season, corresponding to the time of image acquisition. Thereafter, ground data was compared to data derived from image classification. The ground reference label was paired with the remote sensing-derived label for assignment in the error matrix (15). The error matrix showed errors of omission (producer’s accuracy) and commission (user’s accuracy), overall classification accuracy, and a Kappa coefficient. The Kappa coefficient of agreement is a measure of the actual agreement minus chance agreement. The overall classification accuracy is a percentage expressed as the number of correctly classified sample pixels over the total number of sample pixels. This percentage indicated how accurate the classification was with respect to the reference data [17].

The statistical software SPSS and Microsoft Excel were used in analysing data by means of calculations and drawing graphs respectively at a 0.05 level of significance. The mean, and standard deviation were calculated for all the water parameters and results compared to the Department of Water Affairs (DWAF), Food and Agriculture Organisation (FAO), and World Health Organisation (WHO) irrigation water quality standards to assess the suitability of the water for irrigation.

Land use data and water quality data were further analysed using an econometric model shown in Equation (1).

Y = exp (β1 × land1 + β2 × land2 + ⋅ ⋅ ⋅ βi × land i)(1)

where Y means the water quality variables in the study area, α is a constant, and β means the correlation between land use area (%) and water quality variables. When βi > 0, it means that the land use type i has a positive effect on the indicators of water quality. If βi < 0, it means that the land use type i has a negative effect on the indicators of water quality. In addition, the coefficient of determinant (*R*^2^) values were calculated, and these ranged from 0 to 1.0; these can be expressed by percentages ranging from 0 to 100.

## 3. Results and Discussion

In the studied area, there were five main land uses, comprising of woody plants, grass, fresh water, built-up areas, and bare land. The interpretation accuracy of the land use categories reached 92.7%, according to the field survey and random sampling check conducted. Table 2 shows the land cover extents for the sub-catchment areas of five ephemeral ponds and the change statistics.

### 3.1. Land Use Mapping

**Figure 3:**
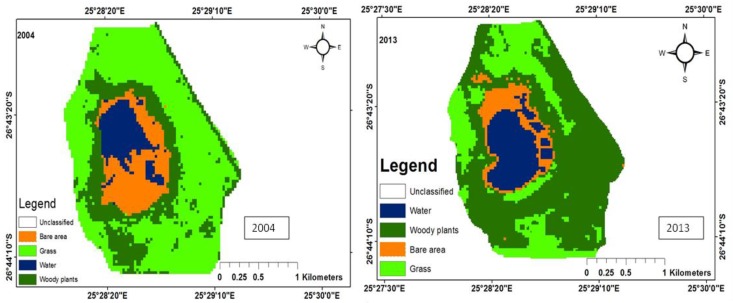
Land use map for Pond A.

**Figure 4:**
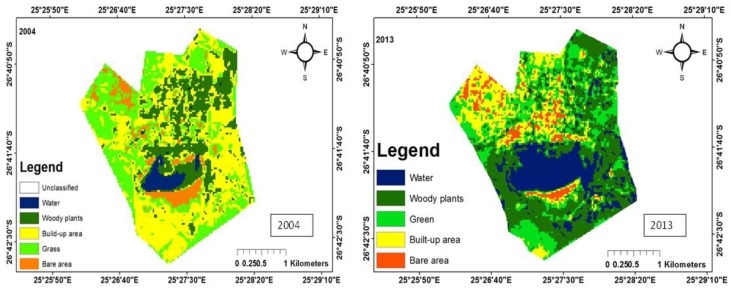
Land use maps for Pond B.

**Figure 5:**
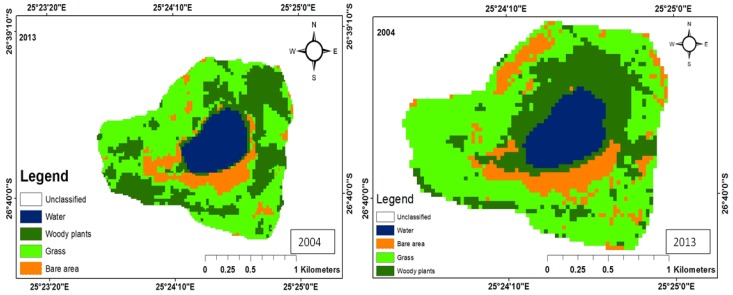
Land use maps for Pond C.

**Figure 6:**
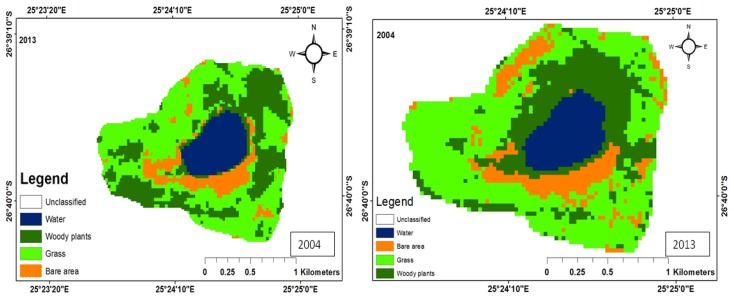
Land use maps for Pond D.

**Figure 7:**
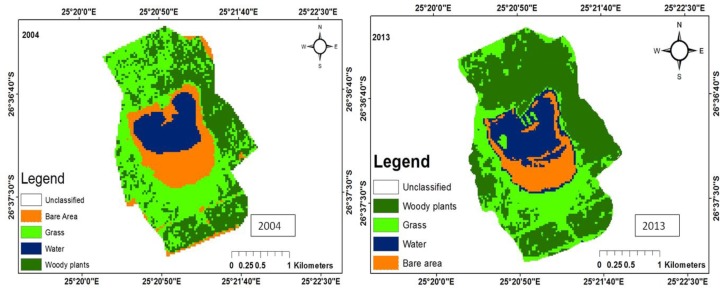
Land use maps for Pond E.

There were five main land uses: woody plants, grass, fresh water, build-up areas, and bare land. The woody trees, grass, fresh water, built-up areas, and bare soil land uses were shaded dark green, green, bluish colour, yellow, and brown, respectively. The woody plants in the study area consist predominantly of grass of the Kalahari Thornveld and shrubs of the Bushveld type. Grass comprises vegetation mainly for grazing. Water refers to ephemeral pond water, patches of water on the soil after rain and ephemeral streams that contain water during periods of high rainfall. Tracks, paths, areas exposed due to fire, over-grazing and parts of the pond that become dry due to low rainfall, evaporation and infiltration, constitute bare areas. Land use change maps for each of the ponds have been shown in Figure 3, Figure 4, Figure 5, Figure 6 and Figure 7, while the trends and statistics for each class are depicted in Table 3. On the basis of these results, trends in land use change are presented in percentage terms for the time period 2004–2013 for woody plants, grass cover, fresh water, bare ground, and built-up areas. However, these trends only apply to the individual pond-sub-catchments. It was not possible to extrapolate these results to report on trends for the entire study area.

#### 3.1.1. Land Use Change for Pond A

Pond A is located far from settlements; hence there is no land use for built-up areas. The woody plant cover in 2004 was 122.853 ha, while that for 2013 was 201.4 ha, representing a 64.0% increase. During the same period, grass cover got reduced by a factor of 21.1%. This reduction in grass cover could be attributed to over-grazing and bush encroachment (Figure 3). There was a 32.8% increase in the area covered by bare area, which could be at the expense of grass (Table 3). In addition, the area covered by water increased by 44.8 ha, which could be attributed to rainfall variability as the area experienced more rainfall compared to 2004 [18]. Immediately after a rain storm, more water collects on the soil surface and the area covered by water for the pond may increase. Low rainfall reduces the area and volume of water in ephemeral ponds and subsequently, the longevity of the ponds. Adeoye [19] attributed reduction in wetland area to climatic variability. Abundant rainfall contributes to rapid growth of vegetation and this would spread to other areas that are devoid of vegetation, which could be the case with the observed 64% increase in woody vegetation.

##### Accuracy Assessment for Pond A

Table 4 shows the error matrix for pond A. The overall Kappa coefficient was 78.9%, this showed a moderate agreement between the correctly classified classes and the reference data. The producer’s accuracy ranged from 76.9% to 88.6%. The lowest value for the user’s accuracy was 77%, while the highest was 90.9%. Grass produced the lowest user’s accuracy of 77.5%. This was due to an inter-class confusion between woody plants and grass. In total, 7.5% of the pixels were wrongly classified as woody plants. Grass and woody plants sometimes form a homogeneous mix and makes spectral separation difficult [20]. Grass and water also showed an inter-class confusion of 2.5% and 11.1%, respectively.

#### 3.1.2. Land Use Change for Pond B

Pond B is located in town hence it has all the classes comprising of woody plants, grass, built-up area, bare area and water. There was an overall increase of 22% and 18.5% in the area covered by woody plants and grass during the study period (Figure 4).

The increase in vegetation cover in 2013 could be attributed to the planting of trees, making of lawns and parks. These practices normally accompany urbanization and development, which started in the area after 2004 as evidenced by the increase in built-up area of 14.1% (Table 2). Since 2004, there has been an increase in the number of Rural Development Plan (RDP) houses in the area. The area covered by water decreased from 379.2 ha to 186.2 ha, which corresponds to the relatively high rainfall in 2004 in this part of the study area (342.8 mm) and the reduction in rainfall (288.2 mm) in 2013. The area created as a result of a reduction of in water was occupied by grass and woody plants.

##### Accuracy Assessment for Pond B

The overall accuracy was 80%. This meant that 80 out of 100 of the data were correctly classified. There was a moderate value for the Kappa coefficient (68.6%). It represented a chance agreement of about 31.4%. All the values for the user’s accuracy were below 90% and could be attributed to confusion between classes (Table 5). The confusion value between the bare area and grass was 16.6%, which could be attributed to the spectral similarities between red soil (0.0974) and dry grass (0.7588) as distinguishing between them became difficult. However, an error of omission occurred between grass and water, where 10% of the pixels for grass was wrongly classified as water. The producer’s accuracy ranged from 73.7% to 100%. However, errors of commission also occurred between grass and water, grass and woody plants, representing 7.4% and 5.6%, respectively.

#### 3.1.3. Land Use Change for Pond C

The sub-catchment area around pond C consisted of woody plants, grass, bare area, and water. No built-up area was identified due to lack of settlements. In 2004, the woody plants covered an area of 110.3 ha and increased to 239.5 ha in 2013, representing a 117.1% (Table 2). Similarly, grass cover increased by 27.3% with a simultaneous decrease in the size of bare area by 91%. During the 9-year period, greater parts of the bare area were occupied by grass and woody plants (Figure 5).

With regard to water, there was an increase in the area occupied (23.0 ha). Greater parts of the bare area were occupied by grass and woody species. This could be attributed to the pond being located on a private farm, where the correct stocking rate is always maintained. Proper pasture management is practiced, including rotational grazing, resulting in fewer bare areas. Moreover, lack of browsers such as goats encourages more woody plants to flourish [21].

##### Accuracy Assessment for Pond C

All the user’s accuracies were high except that for woody plants. Similarly, the producer’s accuracies were above 80%, except for that of water (100, 86.1, 50, and 96.6). This translated into a high overall accuracy of 83.0% (Table 6). This indicated that more than 80% of the data were correctly classified. The Kappa coefficient was 76.3%. However, an inter-class confusion was observed between grass and water. Some grass grew in water and produced an error of 13.8%. Also, a confusion error was observed between the water and woody plants. The branches and leaves of tall trees masked water and therefore resulted in the error of omission. An error of omission of 5.6% was recorded between the woody plants and bare area; this was due to masking of the bare area by tall trees.

#### 3.1.4. Land Use Change for Pond D

The sub-catchment area around Pond D consisted of bare area, water, grass, and woody plants. There was no built-up area due to lack of settlement at the site. The bare area was reduced by a factor of 34.3% from 3153.6 ha in 2004 to 2072.5 ha in 2013. During the same period, the area covered by water changed and this could be due to the marginal difference in the rainfall received in 2004 (342 mm) and in 2013 (288.2 mm). The areas covered by grass in 2004 and 2013 were 1892.5 ha and 1837.1 ha, respectively, amounting to a 3.0% decrease (Figure 6).

#### 3.1.5. Land Use Change for Pond E

Only four land use classes were identified within the sub-catchment area of pond E namely, woody plants, grass, fresh water and bare area. This is due to the absence of settlements at the site. The area covered by woody plants was reduced by a factor of 50.3% between 2004 and 2013. During the same period, grass cover increased by 57.4%. The catchment area is an open field and people from the nearby villages cut the woody plants for fuel. Hence, the vacant areas created were colonized by grass (Figure 7).

The areas covered by freshwater in 2004 and 2013 were 69.4 ha and 130.4 ha, respectively; representing about 88% increase and could be due to rainfall variability. According to the South African Weather Service, Kimberley, on 19 April 2013, daily rainfall of 13 mm was recorded in the study area, preceding the image acquisition date.

##### Accuracy Assessment for Pond E

User’s accuracies of 56.3, 75.7, 85.7, 72.9 and 92.9 were calculated (Table 7). An omission error of 25% was identified between the grass and bare area. Some grass grew on bare area and could not be identified due to the 30 m spatial resolution of Landsat data. This is not accurate enough to identify small field data. An error of omission of 10.3% was recorded between the built-up area and grass. Thatched roof and some buildings have similar spectral values as grass, which makes it difficult to distinguish between the two [20]. A commission error of 10% was observed between the built-up area and water. During heavy rains, water collects in built-up areas and is included in category in the classification system. The overall accuracy showed that about 78% of the data was correctly classified. The Kappa coefficient indicated that the actual agreement with the field data was high compared to that by chance.

### 3.2. Water Quality

Water quality analysis revealed that in general, pond water in the study area has high pH values ranging from 8.6 to 10.6. The electrical conductivity of the water is equally high, ranging from 379 mSm to 780 mSm and above the recommended limits for irrigation water by FAO (0–40) and DWAF (0–70). Besides the physical constituents that affect water quality, there are also some chemicals that impact upon water quality and consequently affect human health. Table 8 shows the chemical characteristics of the pond water during the study period. With respect to the sodium concentration from the study area a mean value of 40.0 mg/L and a range between 37.3 mg/L and 45.7 mg/L was recorded. All the readings were below the limits set by FAO and DWAF. The mean values for calcium and magnesium were 25.53 mg/L and 45.79 mg/L, respectively. There are no recommended values by DWAF. Nevertheless, the relationship between sodium, calcium, and magnesium is expressed as per the sodium adsorption ratio (SARS). The study calculated a SARS value of 6.33, whilst a recommended value of 2 is stipulated by DWAF [22].

In terms of anions, the study was limited to phosphates (PO_4_^—^), nitrates (NO_3_^–^) and chlorides (Cl^−^) for they are important anions with regard to irrigation water quality. Results of the anion concentrations from the sampled ponds with comparison to FAO and DWAF had acceptable levels. However, the microbial analysis revealed very high *E. coli* and total coliform levels above the recommended DWAF and WHO limits. In addition, even though the *E. coli* levels were within the WHO limits, these levels were still above the DWAF acceptable levels (Table 9).

Using the standards provided by DWAF, WHO, and FAO to compare thresholds for water quality, it is noted that some of the values in Table 7 and Table 8 are within acceptable limits. Others however, still fall outside these limits. Given that these values are likely to change even over short periods of time, the ephemeral pond water in the study area can still be harvested for small-scale irrigation purposes.

### 3.3. Relationship between Land Use and Water Quality

The influence of various land cover types on the water quality parameters of the ephemeral ponds was done using regression analysis. Table 10 shows a description of the relationship between the different land cover types and selected water quality parameters. Only those water quality parameters which were above the recommended limits for irrigation were used for the regression analysis.

There was a negative relationship between bare areas and the concentration of Na^+^ ($R^{2}=\;$0.918; *p* < 0.360). The presence of bare areas had significant negative impacts on pond water. This is mainly due to the increase of surface runoff that may coincides with the higher levels of nutrient salts [23], resulting from the agricultural practices and the application of chemical fertilizers [24]. Also, the bare areas had a negative influence onto the electrical conductivity of the pond water. Some studies have found that majority of the bare areas with no or little vegetative cover are likely to contribute to greater runoff [25], thereby degrading the water quality.

Overall, grass cover played a positive role in influencing the water quality for NO_3_^−^, Ec, Cd, and *E. coli* and was negatively related to Na^+^. Sodium concentrations in the ephemeral pond water revealed a negative association with grass cover at (*p* < 0.36; $R^{2\;}$= 0.92) because in the study area, the presence of sodium is mainly through erosion of rocks. The increase in grass cover causes a decrease in sodium as there is less exposure of soil and rocks to erosion. However, grass cover is a predictor for *E. coli* abundance in water with a significant (*p* = 0.006) positive effect on *E. coli* contamination in ephemeral pond water. Grass in the study area is used for grazing and ponds are also used as watering points. When there is abundant grass, a lot of grazing takes place around the pond catchment area; this results in the water being polluted by micro-organisms from the excreta of grazing animals. Hughes et al. [26] found similar relationship between the concentration of *E. coli* and grazing animals, where P, N, and *E. coli* were exported into nearby streams from the wetland as a direct result of disturbance caused by cattle grazing the wetland. Indicator bacteria densities in the stream water were significantly higher when at least 150 herds of cattle were grazing. This is in agreement with Monaghan et al. [27], where N, P, and *E. coli* concentrations increased for spring events, presumably reflecting the effects of pasture grazing that resumed when animals returned to the farm at the end of each winter.

## 4. Conclusions

This study established that in the sub-catchment areas of ephemeral ponds there are many land uses, such as livestock grazing, crop farming, mining, and built-up areas. These activities affect the quality and quantity of ephemeral pond water. In general, the ephemeral pond water quality is affected by land use. The research has found that some ephemeral ponds are of good water quality and can be used to irrigate short, seasoned crops or supplementary dry spells during the cropping season. This can help to improve food security in rural, semi-arid regions.

In this study, all the values recorded for the physical characteristics were higher than the limits recommended by DWAF and FAO. However, the chemical parameters were within the limits except for cadmium. The value calculated for SARS (6.33) was higher than what was recommended by the DWAF [22] water quality guidelines for irrigation. Surprisingly, the recorded *E. coli* measurements satisfied the requirements set by WHO, whilst the total coliform values were higher. The various models have proved successful in determining the effects of land use/land cover change on water quality parameters of ephemeral pond water in the study area. The water could therefore, be suitable for irrigation but should be accompanied by crop, soil, and water management.

Studying the relationship between the proportion of land use types and water quality in ephemeral ponds in the Vryburg area revealed that grass cover was generally positively related to some indicators of water quality (NO_3_^−^, Ec, Cd and *E. coli*) but at the same time, negatively related to Na^+^, while bare areas had a complex influence on the quality of water. An assessment of the impact of land use on water quality is of ultimate importance to all water users. It serves as evidence to decision makers and water planners to take action to control and plan land use in a catchment area. The study highlights the suitability of pond water for irrigation to increase production for rural livelihoods in arid and semi-arid environments. Critical to the use of ephemeral pond water for irrigation is the continuous monitoring of the quality of water and sustainable catchment land use planning.

## Figures and Tables

**Table 1:** Characteristics of the selected ephemeral ponds.

Pond	Location		Area of the Pond (ha)	Depth (m)	Altitude (m)
	Latitude	Longitude			
A	26°32′43″ S	25°03′01″ E	37.8	1.62	1365
B	26°53′52″ S	25°20′06″ E	50.6	2.5	1387
C	26°41′6″ S	25°24′34″ E	17.6	2.3	1351
D	27°16′52″ S	24°46′49″ E	25.3	0.45	1264
E	26°32′52″ S	24°52′28″ E	61.2	2.15	1357

**Table 2:** Water quality parameters, measurements, and units.

Parameter	Measuring Instrument	Unit of Measurement
pH	pH meter	1–14
Electrical conductivity	Conductivity meter	Ms/m
Cations Na^+^, K^+^, Ca^++^, Mg^++^	Variant atomic absorption spectrometry	Mg/L
Heavy metal- Cd,	Variant atomic absorption spectrometry	Mg/L
Anions NO3^−1^, PO4^−3^, Cl^−^	Colorimetry	Mg/L
Total coliform	Membrane filtration	Colony units/100 m
*E. coli*	Agar test	Colony unit/100 m

**Table 3:** Land cover change and trends 2004–2013.

Land Use Class			Woody Plants	Grass	Fresh Water	Bare Areas	Built-Up Areas
Pond A	2004	Area (ha)	122.6	302.8	47.6	178.2	_
	2013	Area (ha)	201.4	238.9	92.1	119.8	_
		% change	64.0	21.1	94.2	32.8	_
Pond B	2004	Area (ha)	168.9	337.0	379.2	107.1	360.5
	2013	Area (ha)	206.1	399.3	186.1	235.7	411.2
		% change	22.0	18.5	50.9	120.1	14.1
Pond C	2004	Area (ha)	110.3	246.4	69.4	196.1	_
	2013	Area (ha)	239.5	313.6	92.4	17.6	_
		% change	117.1	27.1	33.2	91.0	_
Pond D	2004	Area (ha)	1155.5	1892.5	279.0	3153.6	_
	2013	Area (ha)	2223.4	1837.1	0	2072.5	_
		% change	92.1	3.0	100	34.3	_
Pond E	2004	Area (ha)	294.0	238.2	69.39	47.3	_
	2013	Area (ha)	146.3	374.9	130.43	42.1	_
		% change	50.1	57.4	88.0	11.2	_

**Table 4:** Error matrix for classification of Pond A.

Classification Data	Reference Data					
	Bare Area	Grass	Water	Woody Plants	Total	User’s Accuracy (%)
Bare area	28	3	0	0	31	90.3
Grass	5	31	1	3	40	77.5
Water	0	1	10	0	11	90.9
Woody plants	0	0	2	16	18	88.9
Total	33	35	13	19		
Producer’s accuracy (%)	85.9	88.6	76.9	84.2		
Overall accuracy = 85.0%						
Kappa coefficient = 78.9%						

**Table 5:** Error matrix for classification of Pond B.

Classification Data	Reference Data					
	Bare Area	Grass	Water	Woody Plants	Total	User’s Accuracy (%)
Bare area	5	1	0	0	6	83.3
Grass	0	43	5	4	51	79.6
Water	0	4	14	1	19	73.7
Woody plants	1	3	0	18	23	85.7
Total	6	54	19	23	100	
Producer’s accuracy (%)	100	84.3	73.7	78.3		
Overall accuracy = 80.0%						
Kappa coefficient = 68.6%						

**Table 6:** Error matrix for classification of Pond C.

Classification Data	Reference Data					
	Bare Area	Grass	Water	Woody Plants	Total	User’s Accuracy (%)
Bare area	17	0	0	1	18	94.4
Grass	0	31	6	0	37	83.8
Water	0	5	9	0	14	64.3
Woody plants	0	0	3	28	31	90.3
Total	17	36	18	29	100	
Producer’s accuracy (%)	100	86.1	50.0	96.6		
Overall accuracy = 83.0%						
Kappa coefficient = 76.3%						

**Table 7:** Error matrix for classification of Pond E.

Classification Data	Reference Data						
	Bare Area	Built Up Area	Grass	Water	Woody Plants	Total	User’s Accuracy (%)
Bare area	**9**	1	4	0	1	16	56.3
Built up area	1	**22**	2	3	1	29	75.9
Grass	0	3	**24**	0	1	28	85.71
Water	0	3	0	**10**	0	13	72.9
Woody plants	0	1	0	0	**13**	14	92.9
Total	10	30	30	13	15		
Producer’s accuracy (%)	90.0	73.3	80.0	71.4	81.3		
Overall accuracy = 78.0%							
Kappa coefficient = 0.715							

**Table 8:** Water quality parameters.

Sample ID	Na^+^ (mg/L)	K^+^ (mg/L)	Ca^++^ (mg/L)	Mg^++^ (mg/L)	Cd (mg/L)	SAR
A	37.3 ± 2.52	2.5 ± 1.58	20.3 ± 2.7	40.0 ± 1.0	0.02 ± 0.02	
B	38.0 ± 1.0	3.3 ± 0.98	20.3 ± 5.3	38.7 ± 1.5	0.03 ± 0.02	
C	45.7 ± 1.0	2.9 ± 0.65	27.7 ± 1.5	53.0 ± 1.4	0.02 ± 0.02	
D	40.0 ± 1.0	3.8 ± 0.30	29.0 ± 1.0	53.0 ± 1.0	0.03 ± 0.02	
E	39.2 ± 1.0	3.3 ± 0.40	30.3 ± 1.0	44.3 ± 1.5	0.03 ± 0.02	
Food and Agriculture Organisation(FAO)	0–70	0–2	0–20	0–5	Not available	0–3
Department of Water Affairs (DWAF)	0–70		Not available	Not available	0–0.01	0–2

**Table 9:** Microbiological data from water analysis.

Sample ID	*E. coli*	Total Coliform
A	70 ± 68	997 ± 490
B	110 ± 39	888 ± 206
C	8 ± 8.9	2275 ± 250
D	0 ± 0	2277 ± 250
E	201 ± 46	2420 ± 0
**WHO**	0–1000	0–1,000,000
**DWAF**	0–1	Not available

**Table 10:** Regression models for land cover and water quality.

Water Quality Parameter	Model Equation	*R*^2^ Value	*p*-Value
Nitrate (NO_3_^−^)	NO_3_ = −5.317 + 0.026 GRASS + 0.041 FRESHWATER + 0.036 BARE	0.886	0.422
Electrical conductivity (EC)	EC = 182.264 + 6.949 GRASS + 2.857 FRESHWATER − 0.378BARE	0.779	0.576
Sodium (Na^+^)	NA = 71.408 − 0.137GRASS − 0.264 FRESHWATER − 0.146 BARE	0.918	0.360
Cadmium (Cd)	Cd = −0.015 + 0.00GRASS + 0.00 FRESHWATER + 0.00 BARE	0.449	0.849
*E. coli*	*E. coli* = −544.239 + 5.986 GRASS + 4.224 FRESHWATER + 2.735 BARE	1.00	0.006
